# Deployment of check-in nodes in complex networks

**DOI:** 10.1038/srep40428

**Published:** 2017-01-11

**Authors:** Zhong-Yuan Jiang, Jian-Feng Ma

**Affiliations:** 1 School of Cyber Engineering, Xidian University, Xi’an, Shaanxi 710071, China; 2 School of Computer Science and Technology, Xidian University, Xi’an, Shaanxi 710071, China

## Abstract

In many real complex networks such as the city road networks and highway networks, vehicles often have to pass through some specially functioned nodes to receive check-in like services such as gas supplement at gas stations. Based on existing network structures, to guarantee every shortest path including at least a check-in node, the location selection of all check-in nodes is very essential and important to make vehicles to easily visit these check-in nodes, and it is still remains an open problem in complex network studies. In this work, we aim to find possible solutions for this problem. We first convert it into a set cover problem which is NP-complete and propose to employ the greedy algorithm to achieve an approximate result. Inspired by heuristic information of network structure, we discuss other four check-in node location deployment methods including high betweenness first (HBF), high degree first (HDF), random and low degree first (LDF). Finally, we compose extensive simulations in classical scale-free networks, random networks and real network models, and the results can well confirm the effectiveness of the greedy algorithm. This work has potential applications into many real networks.

The advent of complex network^1^,^2^,^3^ theory has had a significant impact on the network and data science^4^ over the course of past 20 years. People’s daily life deeply relies on kinds of artificial networks such as city road networks, highway networks, power grids, communication networks, and virtual networks such as WWW (World Wide Web), social networks, and so on. A wide range of research topics aim to solve the challenges that many real networks face. As discussed in our previous work^5^, a portion of nodes in many complex networks have special functions such gas stations in road networks and highway networks supplying for check-in like services. In air transportation, for the convenience of passengers and resource locations, e.g. maintenance crews, it is very important to locate the hub nodes of an airline^6^. In IT infrastructure, we may want to allocate specific functions to critical nodes or driver nodes^7^, for instance, the nodes that control the Internet traffic in the search for viruses. In interdependent networks (e.g. power grids and communication networks), a fraction of critical nodes may result in the collapse of whole interdependent network^8^, such as the largest blackout of the power gird and the outages of the Internet. In social science, for security purpose, many “inside” agents are need to intercept all communications^9^ in a network of terrorists. In food web^10^, the predation relation can be also considered as check-in like service, and mining the key species whose disappearance may lead to large scale species extinction is a very critical problem. These nodes with special functions can be called check-in nodes, and objects that flow in networks need to finish check-in like services at the check-in nodes. For instance, vehicles often have to pass through gas stations to get gas supplement. Then two aspects of this problem should be considered:

1. Efficient routing strategies. With a portion of predesigned locations (perhaps randomized ones) of gas stations, designing efficient routes for all vehicles is very essential and important to alleviate traffic congestion, save gas fuel and time consumption of drivers. Our work^5^ tried to explore a possible check-in based routing framework for this problem. Definitely, many previous routing optimization methods including the efficient routing^11^, optimal routing^12^, global dynamic routing^13^, incremental routing^14^ and hybrid routing^15^ can be referenced. For simplicity and without loss of generality, here we employ the classical shortest path routing method for path discovery.
2. Optimal deployment of check-in node locations. With a given number of check-in nodes, which positions are the optimal ones that can achieve the highest profits to citizens and governors? To our best knowledge, it is still an open problem in complex network research.

In other words, with minimum number of check-in nodes, we aim to maximize the profits of the whole network in this work. There are several aspects which need to be clarified clearly for this problem:

1. Clear problem definition and evaluation metric. The problem of check-in node deployment should be clear and a metric should be defined to accurately evaluate performance for check-in node deployment methods.
2. Efficient check-in node deployment method. Currently, to our best knowledge, the check-in node deployment method research is open, and there is a lack of deep study.
3. Evaluations. To verify the effectiveness of proposed methods, extensive simulations must be composed in both classical complex network models (e.g. scale-free network model and random network model) and real network models.

In the following section, we will first show the results of this work. Then we introduce the proposed algorithms and the employed network models. Finally, we close this work with a conclusion.

## Results

Here we first define the check-in node deployment problem. Given a network which might be directed or undirected, assuming the shortest path routing protocol is employed, every shortest path between any pair of source and destination must include at least a check-in node to receive the check-in like services. Then the *minimum number of check*-*in nodes* (MNCN) which can guarantee every shortest path including at least a check-in node can be employed to evaluate the performance of a check-in node deployment method.

This problem can be converted into the set cover problem^16^ (see details in Methods section) and solved by employing greedy algorithm^16^ (GA). To compare with GA, other 4 check-in node position selection methods (see details in Methods section) including high betweenness first (HBF), high degree first (HDF), random and low degree first (LDF) are discussed.

Given a set of locations for check-in nodes 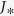, the cover rate of all shortest paths *f* (see details in Methods section) can be employed to evaluate the effectiveness of check-in node location selection methods.

**Figure 1:**
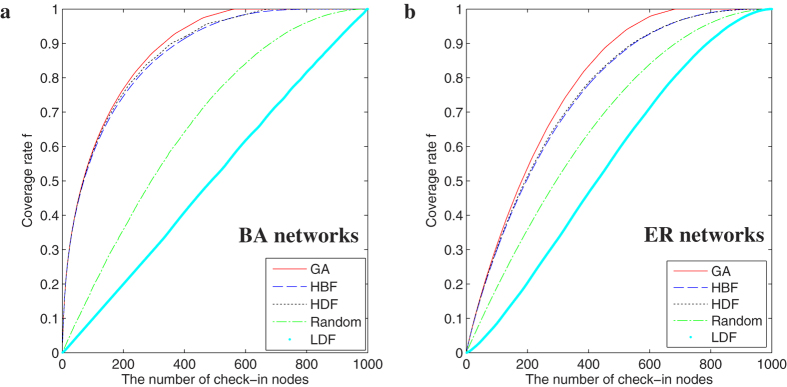
Evolution of cover rate *f* as a function of the number of check-in nodes under the five different check-in nodes selection methods. (**a**) BA Networks; (**b**) ER networks. Network parameters are *N* = 1000, 〈*k*〉 = 8. Each datum is the average of 50 realizations of BA scale-free networks.

We first investigate the evolution of cover rate *f* as a function of the number of check-in nodes in BA^17^ scale-free networks and ER^18^ random networks in Fig. 1(a,b) respectively. One can see that the GA achieves the highest *f*. With the same number of check-in nodes, for instance, 150 check-in nodes in Fig. 1(a,b), *f* under the five methods appears to be GA > HFB > HDF > Random > LDF in both two types of networks. The HBF and HDF appear to be a bit lower than the GA, but very near. The LDF is the worst, because under the shortest path routing, paths trend to pass through the nodes with high degrees. Therefore, with the same number of check-in nodes, the number of the shortest paths that passing through check-in nodes of low degrees is very small, resulting in low *f*. With increasing number of check-in nodes, the *f* increases under the 5 methods. When the number of check-in nodes goes beyond a critical value, the *f* gets its maximum value of 1.0. Then the MNCN can be efficiently achieved and represented by the critical value.

**Figure 2:**
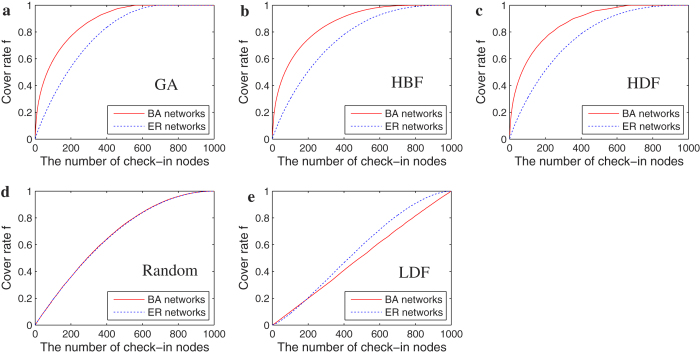
Comparisons of different methods in the two classical networks. (**a**) GA; (**b**) HBF; (**c**) HDF; (**d**) Random; (**e**) LDF. Network parameters are *N* = 1000, and average degree 〈*k*〉 = 8. Each datum is the average of over 50 realizations of BA scale-free networks.

In Fig. 2, we investigate the comparisons of different location selection methods in the two types of classical network models. In Fig. 2(a), based on the GA method, with the same network size and average degree, the robustness of BA^17^ networks appears to be better. It is related to the network structure, and in BA^17^ networks, most of the shortest path pass through a fraction of hub nodes. Meanwhile, the betweenness distribution of all nodes in ER^18^ network is much even, and more check-in nodes are needed. Similarly, under the HDF and HBF methods, the results are very similar to GA. Under the random selection, the effects are almost the same in two network models. In Fig. 2(e), under the LDF method, the ER^18^ network appears to achieve better performance. It is also related to the network structure. The degree distribution of ER^18^ networks is more even than BA^17^ networks.

**Figure 3:**
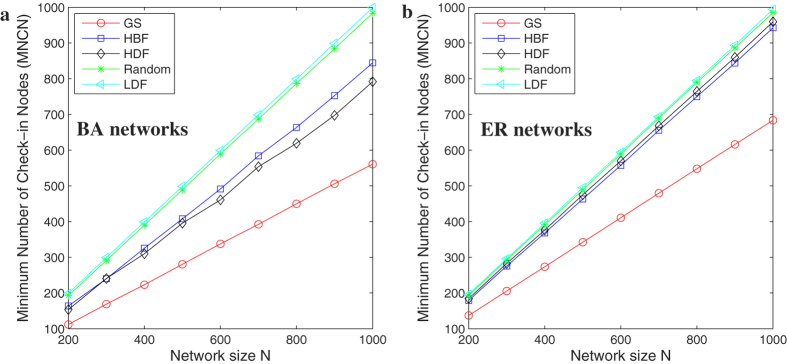
Evolution of MNCN as a function of the network size under the five different check-in nodes selection methods. (**a**) BA Networks of average degree 〈*k*〉 = 8; (**b**) ER networks of average degree 〈*k*〉 = 8. Each datum is the average of 50 realizations of BA scale-free networks.

In Fig. 3, we investigate the evolution of minimum number of check-in nodes (MNCN) under the 5 methods in BA^17^ scale-free networks and ER^18^ random networks of different network sizes. With increasing network size, the MNCN increases. Because the larger the network size, the higher the number of the shortest paths appears, and more check-in nodes are needed. Under all network sizes, the GA method can achieve the lowest MNCN, and it can confirm the effectiveness of GA method.

**Figure 4:**
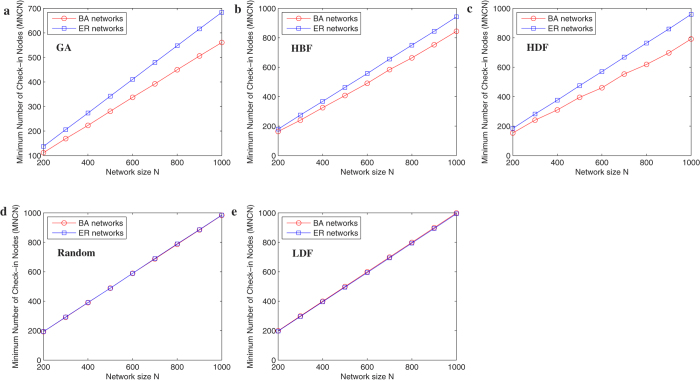
(**a**) Comparisons of different methods in the two classical networks of average degree 〈*k*〉 = 8. (**a**) GA; (**b**) HBF; (**c**) HDF; (**d**) Random; (**e**) LDF. Each datum is the average of over 50 realizations of BA scale-free networks.

In Fig. 4, we investigate the comparisons of all methods in the two network models. We can see that it is very obvious that under the GA, HBF, and HDF, the BA^17^ network models appear to have smaller MNCN, namely higher efficiency than the ER^18^ networks. The effects are almost the same under the Random and LDF methods.

So far, we can say the GA can achieve very good results when compared with all other methods. However, we may want to compare the results with the optimal solution which has been proved to be NP-hard^16^. Here we set network size *N* = 20, average degree 〈*k*〉 = 4. We run the simulations on many BA^17^ and ER^18^ networks on a PC of Intel(R) Core(TM) i5-3470 CPU @3.2 GHz 3.2 GHz, RAM 4.0 GB. In Table 1, the results show that the average MNCN = 9 for both GA and optimal solution in BA^17^ networks, and average MNCN = 11 for both GA the optimal in ER^18^ networks. But the computational cost of the optimal solution is about 4800 and 12000 times more than GA in BA^17^ networks and ER^18^ networks respectively. The results are very amazing, especially for MNCN for both GA and optimal solution. For this special problem, the GA can achieve very good results.

In Table 2, we evaluate MNCN for many real networks which are widely used in previous research papers. One can see that the GA method can efficiently locate the check-in nodes than other 4 methods.

## Discussion

In this work, assuming a portion of nodes were designated as check-in ones to supply check-in services for vehicles or network objects, we aimed to find efficient locations for these check-in nodes to achieve every shortest path including at least a check-in node. By carefully analyzing this problem, we transformed it into a set cover problem which has proved to be NP-complete, and proposed to use the greedy algorithm^16^ to find a cover. To verify the effectiveness of greedy algorithm^16^, we discussed other four heuristic location selection methods including high betweenness first, high degree first, random, and low degree first. To compare these methods, extensive simulations were done in BA^17^ scale-free networks and ER^18^ random networks. We investigated evolution of cover rate as functions of network sizes and average degrees, and found that with increasing network size and average degree the minimum number of check-in nodes which can guarantee every shortest path including at least a check-in node increases. Moreover, we employed these methods into many real network models. All the results can well confirm the effectiveness of the greedy algorithm for set cover problem. We compare the results of the greedy algorithm^16^ with the optimal results, and found that the GA method can achieve better network robustness with low computational cost. The results of this work can be employed for check-in node location selections in many potential real networks. In reality, other factors such as traffic density, source and destination distributions, and routing methods should also be comprehensively considered to efficiently solve the real challenges in complex networks. Moreover, network resilience is a very important topic in network science. In epidemic processes^19^,^20^, it has been found that the epidemic processes are drastically affected by the first two moments of the degree distribution^21^. Can these methods be employed into these network processes and enhance the other network resilience measures? In our future work, we will continue the research topic and share the results soon.

## Methods

### Algorithms

**Figure 5:**
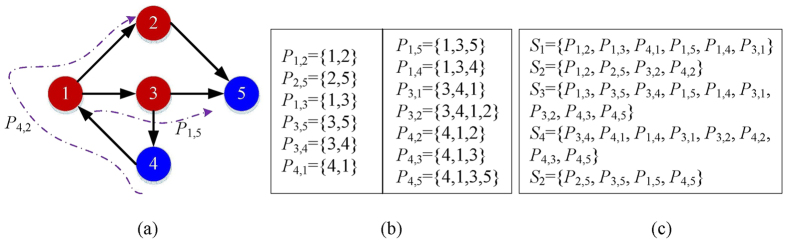
An example for the problem. (**a**) A simple directed network which employs the shortest path routing; (**b**) All shortest paths between all possible source and destination pairs in the network; (**c**) Set *S*_*i*_ denotes the path set in which all paths include node *i*.

As shown in Fig. 5(a), a simple directed network with 5 nodes. The shortest path routing is employed. If many shortest paths exist between a source and destination pair, one of them is used randomly. For instance in Fig. 5(a), the shortest path from node 1 to 5 might be *P*_1,5_ = {1, 2, 5} or *P*_1,5_ = {1, 3, 5}, and we randomly select *P*_1,5_ = {1, 3, 5}. Figure 5(b) shows all the shortest paths in the network.

In order to find the minimum number of check-in nodes, we first collect the shortest paths which pass through a given node. As shown in Fig. 5(c), node 1 has 6 shortest paths including this node, denoted by a set *S*_1_.

In fact, the MNCN problem can be described from another perspective. Given every *S*_*i*_ of node *i* in the network, find the minimum number of sets that can cover all the shortest paths in the network, namely finding a cover *J (J* ⊂ *V*) with minimum |*J*| that can achieve 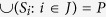, where 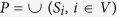 and *V* is the set of all nodes in the network. Then it is converted into the classical Set Cover problem^22^ which has been proved to be NP-complete and can be approximately solved by greedy algorithm^16^ described as follows.

Algorithm 1: Greedy algorithm (GA):

Step 0. Set J = 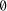.

Step 1. If *S*_*i*_ = 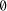 for all *i* then stop: *J* is a cover. Otherwise, find a subscript *j* maximizing |*S*_*j*_| and proceed to Step 2.

Step 2. Add *j* to *J*, replace each *S*_*i*_ by *S*_*i*_ − *S*_*j*_ and return to Step 1.

As shown in Fig. 5, by employing the greedy algorithm, the cover *J* = {3, 1, 2}, namely the minimum number of check-in nodes MNCN equal to |*J*| = 3.

The above greedy algorithm^16^ can find an approximate cover *J*. In the greedy algorithm process, at each step, we find the set which can maximize the number of included shortest paths. Finally, there is a set sequence *J*. However, in real check-in demands, it is not necessary to cover all paths, and we may want to only cover a large portion of them with minimum check-in nodes. Given a subset of *V*, denoted as 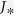, here we employ a cover rate metric to evaluate the covered portion of all the shortest paths, described as


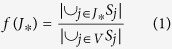


Under the greedy algorithm^16^, when 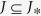, 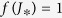. However, the scales of real networks are very large, and it is very difficult to emulate all shortest paths in the network and calculate the set cover. Is there any simple and heuristic algorithm to achieve an approximate cover rate *f* with small number of check-in nodes? Most of real networks can be modeled by the scale-free network model^17^, in which many nodes with the highest degrees are considered as central nodes. Moreover, the betweenness centrality^23^ of a node *v* is defined as the number of shortest paths passing through the node and be used to evaluate the importance of node in the network. Inspired by these heuristic information, in the following parts, we will employ several check-in node selection methods as baselines to compare with the greedy algorithm.

In general, the betweenness of a node directly represents the number of shortest path passing through the node, so the betweenness information based method can be described as follows.

Algorithm 2: High betweenness first (HBF).

Step 0. Sort the betweenness of all nodes in descend order.

Step 1. Given the number of check-in nodes, select the top 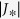 nodes in the descend order.

Step 2. Calculate the 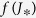.

In HBF, the betweenness of every node must be calculated first. Though the fast algorithm^24^ can be used, it is still consuming huge computation resource especially for large scale networks. Meanwhile, getting the node degree information is relatively simple, and the degree information is also very efficient in evaluating node importance. Moreover, in complex networks, betweenness of a node is strong correlated to its degree. A node of high degree often has large betweenness^23^. Therefore, here we propose a degree based check-in node deployment method.

Algorithm 3: High degree first (HDF)

Step 0. Sort the degrees of all nodes in descend order.

Step 1. Given the number of check-in nodes, select the top 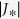 nodes in the descend order.

Step 2. Calculate the 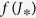.

In HDF, the node degree in employed. Sometimes, the network structure might not be known to us, and no heuristic information can be used. Then the random location deployment mechanism can be simply used.

Algorithm 4: Random

Step 0. Given the number of check-in nodes, randomly select the 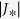 nodes in the network.

Step 1. Calculate the 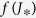.

Opposite to HDF, as discussed in our previous work^5^, if the check-in nodes are selected as the nodes of the lowest degrees, the network traffic capacity^25^ will be remarkably reduced. Here, we assume the nodes of the lowest degrees are set as the check-in nodes, and compare the results with other methods.

Algorithm 5: Low degree first (LDF)

Step 0. Sort the degrees of all nodes in ascend order.

Step 1. Given the number of check-in nodes, select the top 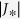 nodes in the ascend order.

Step 2. Calculate the 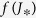.

Moreover, in order to compare the results with optimal solution, here we try to obtain optimal by emulating all possible sets of check-in nodes. Described as follows:

Step 0. Assuming 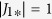.

Step 1. Find all combinations 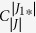.

Step 2. For each combination, if 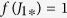 then 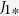 is the result, else 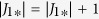, go to Step 1.

### Network models

To verify the effectiveness of above check-in node selection methods, the network structure is the basic. In this work, the used network models include two categories: BA^17^ scale free networks, ER^18^ random networks and real network models.

The BA^17^ scale-free network model which is constructed by two general rules: (1) Growth; (2) Preferential attachment. Starting from *m*_0_ fully connected nodes, a new node with *m (m* ≤ *m*_0_) edges are added to the existing network, and the other end of every new edge is connected to an old node preferentially proportional to the degree of the old node.

Another classical network model is the ER^18^ random graph. The network generation is simple. Initially, beginning with *N* isolated nodes, a pair of nodes is connected by a probability *p*.

## Additional Information

**How to cite this article**: Jiang, Z.-Y. and Ma, J.-F. Deployment of check-in nodes in complex networks. *Sci. Rep.* **7**, 40428; doi: 10.1038/srep40428 (2017).

**Publisher's note:** Springer Nature remains neutral with regard to jurisdictional claims in published maps and institutional affiliations.

## Figures and Tables

**Table 1:** The comparisons MNCN and Computational cost under GA and the Optimal methods in several small network models.

	GA		Optimal	
	MNCN	Computational cost (seconds)	MNCN	Computational cost (seconds)
BA networks	9	0.0136846	9	66.84760
ER networks	11	0.015486	11	190.421226

**Table 2:** The comparisons of minimum number of check-in nodes (MNCN) under different check-in node selection methods in the many real and classical networks.

Type	Name	*N*	*L*	GA	HBF	HDF	Random	LDF
Regulatory	TRN-Yeast-2	688	1079	**123**	675	521	679	678
Trust	Prison-inmate	67	182	**41**	58	62	61	64
	Netscience	1461	5484	**899**	1457	1458	1451	1459
	Leadership	32	96	**19**	24	24	31	31
Food Web	Grassland	88	137	**33**	83	54	86	86
	Seagrass	49	226	**34**	38	41	49	48
	Littlerock	183	2476	**82**	180	145	176	182
	St. Marks	49	223	**33**	38	41	47	48
	St. Martin	45	224	**28**	36	39	42	44
	Ythan	135	597	**57**	133	81	130	134
Biologic Network	E. coli-1	99	212	**63**	89	92	96	97
	E. coli-2	418	519	**103**	414	416	401	413
	S. cerevisiae	688	1209	**145**	675	521	663	678
	Ppi	990	9374	**590**	981	961	980	989
	Neural	297	2345	**193**	280	249	286	295
Electronic Circuits	S208	122	189	**66**	111	112	106	119
	S420	252	399	**133**	233	234	236	250
	S838	512	819	**267**	477	478	494	509
World Wide Wibe	Politicalblogs	1224	19022	**563**	1203	1113	1222	1221
Transposition	Airports	2939	30501	**1101**	2901	2915	2918	2938
Lauguage	Japanese	2704	8300	**502**	2623	2641	2688	2702
BA Model Network	SF2-1	400	797	**166**	201	201	386	399
	SF2-2	400	797	**170**	201	201	383	398
	SF3-1	400	1194	**195**	230	230	388	399
	SF3-2	400	1194	**215**	247	247	386	399
	SF4-1	400	1590	**231**	272	272	389	399
	SF4-2	400	1590	**217**	263	263	388	398
Business	Ownership	141	189	**51**	130	133	137	140
	Wtn61	218	5851	**114**	198	158	212	217

*L* is the number of links of the network.
